# Assessing the translational feasibility of pharmacological drug memory reconsolidation blockade with memantine in quitting smokers

**DOI:** 10.1007/s00213-015-3990-2

**Published:** 2015-06-21

**Authors:** Ravi K. Das, Chandni Hindocha, Tom P. Freeman, Antonio I. Lazzarino, H. Valerie Curran, Sunjeev K. Kamboj

**Affiliations:** Clinical Psychopharmacology Unit, University College London, 1-19 Torrington Place, London, WC1E 6BT UK; Department of Epidemiology and Public Health, University College London, 1-19 Torrington Place, London, WC1E 6BT UK

**Keywords:** Memantine, Smoking cessation, Smokers, Reconsolidation, Addiction, NMDA antagonists

## Abstract

**Rationale:**

Preclinical reconsolidation research offers the first realistic opportunity to pharmacologically weaken the maladaptive memory structures that support relapse in drug addicts. N-methyl D-aspartate receptor (NMDAR) antagonism is a highly effective means of blocking drug memory reconsolidation. However, no research using this approach exists in human addicts.

**Objectives:**

The objective of this study was to assess the potential and clinical outcomes of blocking the reconsolidation of cue-smoking memories with memantine in quitting smokers.

**Methods:**

Fifty-nine dependent and motivated to quit smokers were randomised to one of three groups receiving the following: (1) memantine with or (2) without reactivation of associative cue-smoking memories or (3) reactivation with placebo on their target quit day in a double-blind manner. Participants aimed to abstain from smoking for as long as possible. Levels of smoking and FTND score were assessed prior to intervention and up to a year later. Primary outcome was latency to relapse. Subjective craving measures and attentional bias to smoking cues were assessed in-lab.

**Results:**

All study groups successfully reduced their smoking up to 3 months. Memantine in combination with smoking memory reactivation did not affect any measure of smoking outcome, reactivity or attention capture to smoking cues.

**Conclusions:**

Brief exposure to smoking cues with memantine did not appear to weaken these memory traces. These findings could be due to insufficient reconsolidation blockade by memantine or failure of exposure to smoking stimuli to destabilise smoking memories. Research assessing the treatment potential of reconsolidation blockade in human addicts should focus on identification of tolerable drugs that reliably block reward memory reconsolidation and retrieval procedures that reliably destabilise strongly trained memories.

## Introduction

Substance use disorders (SUDs) involve lasting pathological adaptations in reward learning (Hyman et al. 2006) and motivational (Robinson and Berridge 1993, 2001) memory circuits encoding relationships between environmental stimuli or ‘cues’ and drug availability and reward value, such that these cues motivate drug seeking, craving and relapse when encountered (Kalivas and Volkow 2005). These ‘maladaptive motivational memories’ (MMMs) underlie the long-term hypersensitivity to drug-related stimuli and quiescent susceptibility to relapse in ex-users that typifies addiction (Milton and Everitt 2012).

Extant pharmacotherapies and psychotherapies do not directly modulate MMMs. Some interventions have attempted to inhibit MMMs by augmenting the strength of extinction learning (Kamboj et al. 2012; Kamboj et al. 2011), but these have met with limited success. This is likely because extinction does not directly weaken MMMs and is therefore only as effective as the continued capacity of extinction memories to inhibit prepotent MMMs. Brief extinction learning is largely insufficient to compete with long-established maladaptive memories, and the effects of putative consolidation enhancers (Das and Kamboj 2012) are too modest to redress this imbalance in memory strength.

An alternative approach that aims to directly weaken MMMs during memory reconsolidation has inspired much interest in the past decade. ‘Reconsolidation’ is a term used to refer to the process of *destabilisation* and subsequent *restabilisation* of memory traces upon recall (Nader et al. 2000) allowing prescient new information to be incorporated into existing memory traces to keep them up-to-date (Lee 2009). The restabilisation of appetitive memories is dependent upon cascades of de novo protein synthesis via activation of the N-methyl-D-aspartate receptor (NMDAR) and the transcription factor, *Zif268* (Lee and Hynds 2013). As such, antagonising NMDARs (Lee and Everitt 2008; Milton et al. 2008a), interfering with gene transcription or inhibiting protein synthesis (Blundell et al. 2008; Nader et al. 2000) following memory destabilisation can greatly weaken memory traces in laboratory animals. Reconsolidation research therefore offers the first credible opportunity for directly weakening MMMs and potentially reducing long-term relapse rates in addicts (Milton and Everitt 2012).

Despite the promise of this approach, there are major obstacles hindering its application in human addicts. Primary amongst these are the sensitivity of memory destabilisation to parameters of retrieval (Lee 2009; Pedreira et al. 2004), age and strength of the retrieved memory and the lack of compounds available for human use known to block restabilisation. Length of the memory retrieval session, number of cue presentations and similarity of retrieval to learning all constrain destabilisation of memories, determining the switch between the mutually exclusive (Merlo et al. 2014) processes of reconsolidation and extinction (Osan et al. 2011; Pérez-Cuesta and Maldonado 2009; Suzuki et al. 2004). The effect of these parameters may all be mediated by their impact on the generation of mismatch, or prediction error, during retrieval (Sevenster et al. 2013), which is necessary for memory destabilisation.

Older, more robust memories are generally less susceptible to destabilisation when retrieved (Robinson and Franklin 2010). Although paradigms involving instrumental responding may involve hundreds of action-outcome pairings and still show reconsolidation blockade (Lee et al. 2006; Milton et al. 2008a; Milton et al. 2008b), in human addictions such as smoking and alcoholism, MMMs are formed over tens or hundreds of thousands of action-reinforcement pairings (e.g. a 20-per-day smoker, taking 15 drags per cigarette, for 2 years = 146,000 reinforced inhalation actions) and should therefore be considered extremely robust. Despite highly promising findings using cue exposure following destabilisation of cue-drug memories in heroin users (Xue et al. 2012), the destabilisation of cue-smoking or cue-drinking MMMs has not yet been shown in humans and, given their training history, may not even be possible.

We recently demonstrated that of two classes of drugs that show translational promise for blocking MMM reconsolidation, NMDAR antagonists and β-blockers, NMDAR antagonists display much more robust effects than β-adrenergic antagonists (Das et al. 2013). Only one study (Saladin et al. 2013) has attempted to translate these preclinical findings into humans. This attempt to use propranolol to disrupt reconsolidation in cocaine-dependent individuals found the relatively modest effects predicted from the meta-analytic findings. Although short-term effects of propranolol were found, lasting effects (which would be expected if reconsolidation were interrupted) were not.

To date, no research exists examining the potential of interfering with MMM reconsolidation by NMDAR antagonism in human addicts. While NMDAR antagonists interfere robustly with memory restabilisation in animals, they are also often dissociative, psychotomimetic (Muetzelfeldt et al. 2008) and neurotoxic (Fix et al. 1993), limiting their utility in human addicts. Of the very limited class of NMDAR antagonists available for human use that do not produce these effects, memantine is a potentially promising reconsolidation-disrupting agent in humans, with translational potential supported by the observation that it interferes with MMM restabilisation in rodents (Alaghband and Marshall 2013; Popik et al. 2006).

Memantine is very well tolerated and does not exhibit the side effects of other NMDAR antagonists at low doses (Parsons et al. 1999). However, findings with memantine in reconsolidation are inconsistent and species-dependent (Samartgis et al. 2012). Further, due to the lack of human MMM reconsolidation research and the novel pharmacodynamics of memantine at the NMDA receptor (Rammes et al. 2008; Xia et al. 2010), there is very little information upon which to base an experimental memantine dose. Meta-analysis suggests a non-linear dose-response effect of MK-801 on reward memory reconsolidation blockade, with low doses exhibiting greater efficacy than moderate doses (Das et al. 2013). Although memantine has lower NMDAR affinity than MK-801 (Rammes et al. 2008), low doses of the drug have been found to induce memory impairments in rats, with higher doses generating an intolerable side effect profile (Creeley et al. 2006). In cloned human receptors, memantine in high concentrations antagonises both NMDARs and nicotinic acetylcholine receptors (Maskell et al. 2003). This lack of specificity impedes the attribution of any observed effects to glutamatergic reconsolidation systems.

Human research in smokers has utilised up to 40 mg memantine. At this dose, memantine produces significant dizziness, light-headedness, detachments from reality and temporal distortion and prevents the ‘buzz’ smokers experienced following a cigarette (Jackson et al. 2008). Appropriate dosing should aim to minimise this side effect profile, maximise the NMDAR specificity of memantine and avoid the potential dip in efficacy of moderate-dose NMDA antagonism.

Given the great translational potential of NMDAergic human MMM reconsolidation blockade, but paucity of research therein, we sought to establish proof-of-principle that memantine could interfere with the reconsolidation of MMMs in quitting tobacco smokers, a prototypical addicted population, to reduce relapse and cognitive measures of MMM strength.

Instead of conducting a premature and costly clinical trial, we employed an experimental medicine approach that capitalised on participants’ voluntary quitting. Following memantine combined with smoking memory reactivation, we sought evidence of NMDAR-mediated blockade of MMM reconsolidation, as indicated by longer relapse latency, fewer cigarettes smoked at follow-up and reduced dependence score (primary outcomes) and reductions in cue reactivity and an attentional bias measure of smoking cue motivational salience (secondary outcomes). Taking account of the aforementioned issues with dosing, a relatively low (compared to previous studies) dose of 10 mg memantine was selected.

## Materials and methods

### Participants and design

Based on a conservative effect size of *r* = 0.35, power calculation for 0.8 power at α = 0.05 yielded a required *N* of 57. Assuming minimal attrition, 59 smokers were recruited via internet advertisement. Inclusion criteria were ages >18<65, scoring >4 on the Fagerstrom Test of Nicotine Dependence (FTND) (Heatherton et al. 1991), smoking >10 cigarettes every day, a strong desire to stop smoking and intending to within 3 months and willingness to make a serious attempt at sustained abstinence that could be timed with the first study day. Exclusion criteria were current/history of mental health or neurological conditions, concurrent addiction to any other substance, use of any illicit drug more than once per week, use of ketamine more than once per month, pregnancy or breastfeeding and compromised renal or hepatic function. Of the participants randomised to a group, four did not attend the second study session and were lost to all further follow-up. We utilised an intention-to-treat approach such that all participants randomised contributed data to the statistical analyses. All procedures were approved by the UCL ethics committee.

A randomised, double-blind, placebo-controlled design assessed the effects of memantine on MMM reconsolidation. Participants were randomly assigned to the following groups: brief reactivation of smoking MMMs with memantine (MEM+ REACT, *N* = 19), reactivation of smoking MMMs with placebo (PLA +REACT, *N* = 20) or memantine without reactivation of smoking MMMs (MEM no REACT, *N* = 20). Drug was 10 mg oral memantine hydrochloride (Namenda) formulated in opaque gelatine capsules with lactose filler. Placebos were matched lactose-only capsules.

### Tasks and apparatus

#### Visual probe

A visual probe task motivational salience of smoking cues on *day 8* of the study. As motivational salience is thought to be a product of reward learning, weakening MMMs should result in reduced salience of smoking cues and reduced attentional bias to these cues. The task used two *types* of image pairs: smoking pictures paired with composition-matched neutral images (*n* = 20) or control neutral-neutral (*n* = 20) pairs. The task and the images are described completely in the paper from which they were taken (Mogg et al. 2003). Image pairs appeared for 500 or 2000 ms and were replaced by probes either contralateral or ipsilateral to the target (smoking-related). Trial presentation was counterbalanced for duration, target side and probe/target congruence.

#### Smoking, subjective and physiological assessments

Primary outcomes of nicotine dependence and continuous level of smoking were assessed with the Fagerstrom Test of Nicotine Dependence (FTND) (Heatherton et al. 1991) and a daily online ‘smoking diary’ starting 1 week prior to *day 1* (baseline) and continuously from then up to 3 weeks following *day 8*. The smoking diary for each participant was checked daily, and they were contacted by telephone to remind them to fill it out if entries fell more than 24 h behind. The subjective assessment battery consisted of the 10-item Questionnaire on Smoking Urges (QSU) assessing craving (Tiffany and Drobes 1991); Mood and Physical Symptoms Scale (MPSS) (West and Hajek 2004), assessing withdrawal-related symptoms; Spielberger State-Trait Anxiety Index (STAI) (Spielberger et al. 1970), assessing state and general anxiety levels; Barratt Impulsiveness Scale (BIS), assessing levels of trait impulsivity (Patton and Stanford 1995); Beck Depression Inventory (BDI), measuring depressive symptomatology (Beck et al. 1988); Temporal Experience of Pleasure Scale (TEPS) assessing hedonic processing (Gard et al. 2006); and Perceived Social Support scale, friend (PSS-FR) and family (PSS-FA) (Procidano and Heller 1983) versions, assessing supportive structures surrounding participants. This battery of tests was included based upon previous research showing that these constructs are important predictors of smoking cessation success (Mermelstein et al. 1986; Powell et al. 2010; Powell et al. 2004) in order to better assess changes that were specifically manipulation-related.

Single-item 100-mm VAS scales were used immediately pre- and post-video to assess cue-induced craving during reactivation. These scales required participants to mark down the strength of their urge to smoke and were anchored ‘No urge at all’ and ‘Strongest Urge Ever’. They were used in place of the QSU as cue reactivity measures to provide rapid momentary assessment, minimise completion time and interference with electrophysiological measures. Such scales have high convergent validity with the QSU (West and Ussher 2010). Eye movement data during the visual probe were acquired with a desktop-mounted Eyelink 1000 eye tracker (SR Research, Ontario, Canada) with participants’ heads stabilised 70 cm from the 1024 × 768 monitor used to display all computer tasks. Blood pressure was measured with an Omron 708-BT electronic blood pressure cuff (Omron, Japan) and skin conductance and heart rate variability (HRV) were recorded using an Equivital EQo2 Lifemonitor belt and sensor with auxiliary skin conductance electrodes (Hidalgo, Cambridge, UK) attached to the medial phalanges of the participants’ left hand with AMBU white sensors.

#### Procedure

The first study session (day 1) was arranged to fall on participants’ target ‘quit day’. Participants filled out the daily online smoking diary for the week preceding *day 1*. Participants were asked to refrain from smoking for 1 h prior to the beginning of *day 1*, to fast for at least 3 h and to avoid the use of alcohol or any drug in the 24-h preceding sessions.

#### Day 1 (baseline/intervention)

Participants took the capsule memantine or placebo capsule immediately after informed consent. Breath carbon monoxide (CO) was then measured with a Micro+ CO meter (Bedfont, UK). Participants then completed the subjective assessment battery and a saccade/antisaccade task and effort-based reward task, which are not reported here. Participants then waited until 3.5 h had elapsed since they took the capsule, based on oral memantine reaching peak plasma concentrations at 3–7 h post-administration, to coincide peak concentrations with the reconsolidation window (at ~4 h post-pill). After the break, participants were fitted with ECG and skin conductance electrodes and began the retrieval procedure.

#### MMM/control retrieval

Smoking MMM reactivation stimuli were boxes containing physical smoking cues (Marlboro cigarettes, lighter and ashtray) and six 30-s validated video clips depicting people smoking in various locations with smoking paraphernalia. Non-reactivation stimuli consisted of six similar 30-s clips that did not depict smoking or smoking-related cues (Tong et al. 2007) and a box containing numbered cards and a pencil. The boxes and videos were labelled so that the experimenter was blind to their contents.

Participants were given the relevant in vivo stimuli box and informed that they would receive on screen instructions before watching some video clips. Prior to starting the videos, a 5-min heart rate baseline, blood pressure and single-item craving was recorded.

**Fig. 1:**
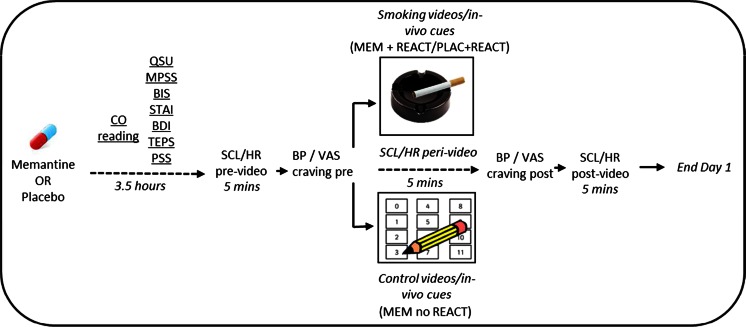
Schematic of testing order on study day 1. Day 8 followed an identical testing order, without drug administration or waiting period. The visual probe was performed as the last task on this day

Before the videos, the on-screen instructions read as follows: “*In front of you is a box*, *please open the box now and take note of its contents and leave it open until you are told to close it. In the box there is* [a lighter, cigarettes and ashtray/ a deck of numbered cards, paper and a pencil]. *These are for a task that you may be required to perform after watching a series of short videos. This task will be performed outside of the building*, *with a different experimenter. When you are watching the videos*, *try to imagine being in the depicted scenes as much as possible*, *imagining the sights*, *smells*, *sensations and sounds as if they were really there. You will be told whether or not you need to complete the task after the videos finish*.” The videos then played and after their conclusion participants were informed that they would not complete the task, to close the box and alert the experimenter. Another blood pressure and single item VAS craving measure was then recorded, and a 5-min period of heart rate post-video collected. To ensure engagement with the reactivation procedure, participants were then given space to write a summary of the procedure that had happened, what they had seen in the videos, what the videos and box reminded them of and what they believed the task involved. Finally, participants guessed whether they received drug or placebo. A schematic of the testing order and timing on day 1 is shown in Fig. 1.

#### Day 8 (test)

Participants returned to the study centre and a carbon monoxide reading was taken. They then completed the subjective assessment battery and in vivo/video ‘reactivation’ procedure along with heart rate, blood pressure and craving measures to assess changes in cue reactivity. Finally, the visual probe task was completed and this concluded testing. Participants continued to fill out the smoking diary for three more weeks. Participants were reimbursed at a rate of £8 per hour. To incentivise compliance with smoking diary completion following day 8, payment was split between day 8 and 3 weeks post-day 8, with half at each time point.

Follow-up measures were completed by telephone at ~3-month intervals for up to 1 year. All participants were followed for at least 3 months and if their smoking returned to pre-study levels, they were followed up no further. If participants became uncontactable, their scores on smoking-related primary outcomes were returned to baseline level.

#### Statistical approach

All data analysis was performed using IBM SPSS version 21 for Windows. Group assignment was only unblinded after analysis was completed. Data were checked for normality, homogeneity of variance and sphericity by inspection of histograms and z-scored skewness/kurtosis, Levene’s test and Mauchly’s test, respectively. Any outliers more than 3 standard deviations away from the sample mean for that variable were replaced with a score falling 3 standard deviations from the mean. Data were square root or log transformed where skewed. If this did not normalise the distribution, non-parametric equivalents of tests were used as appropriate. Descriptive statistics represent untransformed data, unless stated otherwise, in order to aid interpretation of results. Where homogeneity of variance was violated in one-way ANOVA, Welch’s F test is reported. Where sphericity was violated, the Huynh-Feldt correction was applied to the degrees of freedom and significance levels. Uncorrected degrees of freedom are reported here, with corrected *p* values. For single time-point measurements, one-way ANOVA was used to assess group differences and for repeated measurements, mixed ANOVA with a between-subjects factor of group was used. Significant main effects and interactions in omnibus ANOVAs were investigated with independent or paired sample *t* tests on marginal means, where appropriate. Survival analysis on relapse latency was performed using Cox regression stratified by group. pairwise comparisons on k >2 effects in omnibus ANOVAs were Bonferroni-corrected.

## Results

To maintain clinical relevance, we employed an intention-to-treat analysis with dropout coded as treatment failure and scores on primary outcomes returned to baseline. For dropouts, visual probe data were imputed using the estimation maximisation method as Little’s test indicated that data were missing completely at random (χ^2^(69) = 77.094, *p* = 0.236). Descriptive statistics for baseline measures are given in Table 1. The groups differed only on craving, with higher craving in MEM no REACT compared to PLAC + REACT and marginally on BIS, with lower scores in PLAC + REACT. All analyses were run with and without these scores as covariates, and no substantive difference was found, so reported statistics represent those without covariates included. Compliance with filling out the smoking diary was very good in all groups, with over 90 % days completed by all participants and no more than 1 day consecutively omitted by any participant that completed treatment. Mean per day smoking was therefore calculated for the baseline and post-quit periods.

**Table 1:** Descriptive statistics (mean ± standard deviation) and associated significance of tests of group means for smoking and mood variables at baseline

	MEM no REACT (*N* = 20)	PLAC+REACT (*N* = 20)	MEM+REACT (*N* = 19)	ANOVA significance
Age	27.45 ± 6.91	28.35 ± 7.04	29.32 ± 9.9	0.769
Years in Education	15.33 ± 1.98	16.45 ± 3.02	15.47 ± 2.9	0.304
Pre Quit FTND	5.4 ± 1.05	5.6 ± 1.05	5 ± 0.75	0.15
Pre Quit Cigarettes Per Day	14.2 ± 4.27	14.45 ± 3.33	14.53 ± 3.2	0.958
Years smoking	11.15 ± 5.78	10.75 ± 6.59	11.24 ± 7.36	0.97
Pre Quit CO (ppm)	7.84 ± 5.7	9.8 ± 4.4	11.95 ± 6.5	0.081
Number previous quits	2.11 ± 1.2	2.45 ± 1.7	2.53 ± 2.25	0.736
Previous longest quit (days)	188.58 ± 358.3	121.85 ± 249.67	169.16 ± 493.95	0.877
Last cigarette (mins)	833.7 ± 184.51	248.45 ± 294.94	204.16 ± 227.08	0.416_w_
QSU Baseline	37.75 ± 14.93	25.45 ± 10.23	32 ± 12.88	0.014*
MPSS Mood	0.96 ± 0.69	0.63 ± 0.35	0.72 ± 0.48	0.185_w_
MPSS Urge Frequency	2.15 ± 1.31	1.75 ± 1.07	1.95 ± 1.13	0.563
MPSS Urge strength	2.40 ± 1.31	1.90 ± 1.21	1.63 ± 0.68	0.098
BIS Total	69.65 ± 11.40	61.6 ± 12.51	69.63 ± 11.28	0.053
STAI	36.95 ± 11.83	32.6 ± 7.87	33.05 ± 6.91	0.266
BDI	2.1 ± 2.51	2.1 ± 1.83	2.26 ± 1.48	0.958
TEPS Anticipatory	4.56 ± 0.79	4.7 ± 0.58	4.59 ± 0.77	0.815
TEPS Consummatory	4.59 ± 0.84	4.74 ± 0.8	4.72 ± 0.79	0.818
PSS-FR	14.85 ± 4.92	15.3 ± 3.05	14.84 ± 3.88	0.919
PSS-FA	11.75 ± 6.23	12.6 ± 5.92	10.53 ± 6.34	0.576

All tests were one-way ANOVA except where marked with a subscript W, indicating that Welch’s ANOVA was used due to heterogeneity of variance. *Ppm* parts per million;*significant at *p* < 0.05

### Changes in smoking behaviour

Initial analysis of follow-up data showed that no change in smoking status occurred in any participants after the first 3-month follow-up and that the majority of participants had returned relapsed by this time. As such, the first 3-month follow-up only is included in the subsequent statistical analyses. Mixed 2 (day 1, day 8) × 3 (group) ANOVA found a reduction in breath CO between days in all groups [time main effect F(1,56) = 141.822, *p* < 0.001, η_p_^2^ = 0.717] but no group or group × day interactions. A 3 (baseline week, post-quit week, 3 months post-quit) × 3 (group) ANOVA showed a reduction in mean daily number of cigarettes smoked [time main effect F(1,56) = 10.586, *p* = 0.002, η_p_^2^ = 0.159] from the pre-to-post quit week [*t*(58) = 11.91, *p* < 0.001, *r* = 0.84], and a rebound from post-quit to3 months post-quit [*t*(58) = 5.49, *p* < 0.001, *r* = 0.58] (descriptive statistics for these data are given in Table 2). Smoking levels 3 months post-quit smoking were still lower overall than at baseline, however [*t*(58) = 6.04, *p* < 0.001, *r* = 0.62].

**Table 2:** Descriptive statistics of smoking outcomes across the experimental groups

Group	MEM no REACT	PLAC+REACT	MEM+REACT
Day 8 N Not smoking/still smoking	7/13	11/9	6/13
Day 8 N smoking less/smoking as much	15/5	18/2	12/7
Pre Quit week cigarettes per day	14.2 ± 4.27	14.45 ± 3.33	14.53 ± 3.2
Post-quit week cigarettes per day	4.13 ± 4.48	3.2 ± 3.93	6.66 ± 6.3
3 month cigarettes per day	7.91 ± 6.45	8.55 ± 7	10.44 ± 5.45
Mean relapse latency (days)	22.7 ± 78.97	95 ± 151.44	47.32 ± 110.85
Median relapse latency (days)	1 ± 3.33	5 ± 10	1 ± 1.88
N guessing drug	1	9	9
N guessing placebo	7	4	6
N guessing don’t know	12	7	4

Mean and median relapse latency are given to illustrate that, although a few participants successfully quit for long periods (affecting the mean latency statistic), most relapsed soon after quitting

### Survival analysis

**Fig. 2:**
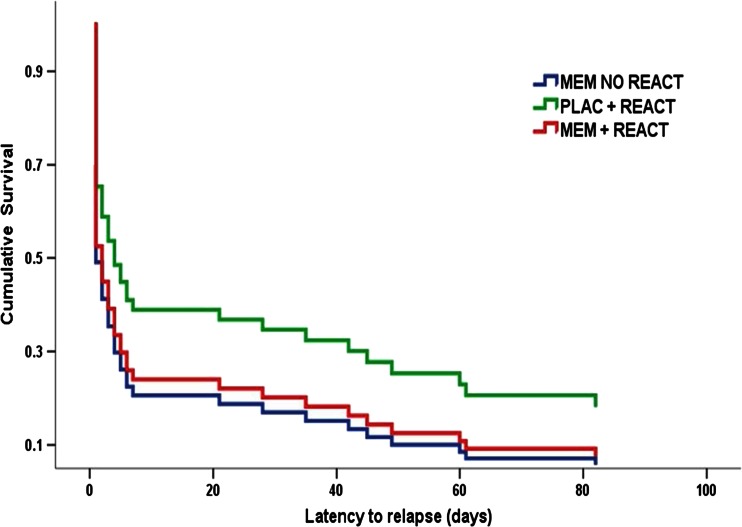
Survival curves for relapse latency by experimental group, adjusted for craving. Curves are censored at 85 days as there was no change in relapse status after this time point

Cox regression assessed relapse latency (descriptive statistics of relapse data are shown Table 2) across the three groups. In total, 8 cases were censored due to not having relapsed by their final follow-up [MEM no REACT *n* = 1; PLAC + REACT *n* = 5, MEM+REACT *n* = 2]. Adding group to the basic regression model did not significantly improve model fit [-2LL change = 4.435, χ^2^ (2) = 0.109]. Contrasts between regression slopes for each group found no significant differences between any group (all *p*s > 0.1) and entering craving and baseline dependence as covariates did not significantly affect the model (*p*s > 0.1). A survival plot for these data is shown in Fig. 2. Curves are plotted to 85 days as there was zero variance in relapse status up to 365 days in all participants who were abstinent at this time point.

### Craving

Reductions in QSU craving were seen between days 1 and 8 [time main effect F(1,56) = 19.333, *p* < 0.001, η_p_^2^ = 0.257]. A main effect of group was also observed [F(2,56) = 5.788, *p* < 0.001, η_p_^2^ = 0.171], driven by higher craving on both days in MEM no REACT than PLAC+REACT [*t*(38) = 3.39, *p* = 0.004, *r* = 0.48]. *Day 1* QSU score was positively correlated with *day 8* smoking levels [*r*(59) = 0.367, *p* = 0.004] and attentional bias to smoking images [*r*(59) = 0.309, *p* = 0.017] and predicted 3-month FTND score [*r*(59) = 0.5, *p* < 0.001] and shorter relapse latencies [*r*(59) = −0.367, *p* = 0.004]. Overall, there were no group differences in FTND [F(2, 56) = 0.569, *p* = 0.569, η^2^ = 0.02] or cigarettes smoked per day [F(2, 56) = 0.355, *p* = 0.703, η^2^ = 0.01] at 3 months post-quit or subsequently. There was thus no evidence for ‘lagged’ intervention effects, as have previously been observed with reconsolidation-based interventions (Soeter and Kindt 2015).

The MPSS showed decreases in urge to smoke frequency [time main effect F(1,56) = 6.393, *p* = 0.014, η_p_^2^ = 0.102] and strength [time main effect F(1,56) = 4.778, *p* = 0.033,η_p_^2^ = 0.079] in all groups from *day 1* to *day 8*, but no change in mood [time main effect F(1, 56) = 2.239, *p* = 0.14, ns]. No group or group × time effects were observed.

### Smoking cue reactivity

Descriptive statistics for cue reactivity measures are presented in Table 3. A 2 (day 1, day 8) × 2 (pre-video, post-video) × 3 (group) mixed ANOVA showed decreased craving in all groups from days 1 to 8 [time main effect F(1,56) = 22.114, *p* < 0.001, η_p_^2^ = 0.283] and a trend for an increase in craving pre- to post-video on both days [pre-post main effect F(1,56) = 3.017, *p* = 0.088, η_p_^2^ = 0.051]. No effects of group or interactions were found. No effects of the video, group or study day were observed for systolic blood pressure (all *F*s < 2.3, *p*s> 0.1). For diastolic blood pressure, a main effect of group was observed, with lower diastolic blood pressure in MEM no REACT than PLAC + REACT and MEM + REACT overall [group main effect F(2,56) = 3.728, *p* = 0.03, η_p_^2^ = 0.117], but with no day, pre-post effects or interactions (all Fs < 1, *p*s> 0.45).

**Table 3:** Descriptive statistics for measures of smoking cue reactivity

	MEM No REACT			PLAC+REACT			MEM+REACT		
	Pre	Peri	Post	Pre	Peri	Post	Pre	Peri	Post
SCL Day 1 (μS)	3.16 ± 2.91	3.58 ± 3.62	4.5 ± 4.05	3.84± 2.16	4.70 ± 2.22	5.54 ± 2.68	4.47 ± 2.83	5.13 ± 3.18	6.05± 3.61
SCL Day 8 (μS)	3.57 ± 2.6	4.04 ± 3.12	4.85 ± 3.64	3.57 ± 1.86	3.6 ± 2.06	4.23 ± 2.53	4.27 ± 2.64	4.39 ± 2.53	5.42± 2.87
HRV day 1 (SDRR)	10.14 ± 6.57	6.86 ± 4.8	8.34 ± 4.34	6.87 ± 3.81	5.58 ± 4.05	7.46 ± 4.34	7.05 ± 3.75	6.08 ± 4.13	7.69± 4.35
HRV day 8 (SDRR)	9.53± 5.42	7.86 ± 5.39	10.64 ± 5.66	7.92 ± 4.18	7.25 ± 7.37	7.79 ± 5.57	8.17± 4.70	5.69 ± 3.70	6.71± 3.97
Craving day 1	48.3 ± 14.87	–	46.55 ± 22.75	41.73 ± 27.93	–	49 ± 27.18	48.5 ± 25.77	–	53.7 ± 31.78
Craving day 8	34.7 ± 25.95	–	35.19 ± 24.64	22.55 ± 17.47	–	25.8 ± 22.75	29.42 ± 26.75	–	39.37 ± 34.12
Systole day 1 (mmHg)	106.9 ± 11.9	–	105.1 ± 9.35	110.25 ± 15.21	–	108 ± 14.70	108.84 ± 13.12	–	109.9 ± 14.87
Diastole day 1 (mmHg)	65.25 ± 6.91	–	66.4 ± 5.932	71.3 ± 12.13	–	71.7 ± 10.87	70.74 ± 9.83	–	71.95 ± 10
Systole day 8 (mmHg)	103.2 ± 10.13	–	101.55 ± 8.44	109.35 ± 12.59	–	107.5 ± 13.52	109.47 ± 15.77	–	110.4 ± 15.42
Diastole Day 8 (mmHg)	63.9 ± 8.12	–	65.3 ± 4.47	71.85 ± 10.25	–	70.45 ± 9.47	71.11 ± 11.13	–	71.37 ± 9.91

Data represent means ± SD

*SCL* skin conductance level, *HRV* heart rate variability, *μS* micro Siemens, *SDRR* standard deviation of R-R intervals, *mmHg* millimetres of Mercury

A 3 (time: pre-video, peri-video, post-video) × 2 (day: day 1, day 8) × 3 (group) ANOVA on HRV data (calculated at standard deviation of R-R intervals; SDRR) found a quadratic main effect of time [F(2, 112) = 11.925, *p* < 0.001, η_p_^2^ = 0.176], with a reductionin HRV pre-to-peri video [*t*(58) = 4.262, *p* < 0.001, *r* = 0.49] and an increaseperi-to-post video [*t*(58) = 3.938, *p* = 0.001, *r* = 0.46]. A trend-level time × day × group interaction was also found [F(4,112) = 2.354, *p* = 0067, η_p_^2^ = 0.078] indicating that the reduction of HRV from pre-to-peri video was significant only in the MEM no REACT group [*t*(58) = 2.917, *p* = 0.015, *r* = 0.36].

Skin conductance data (calculated as mean level of conductance in microSiemens) also showed a time main effect [F(2,112) = 47.211, *p* < 0.001, η_p_^2^ = 0.457], with conductance increasing in a linear fashion from pre-to-peri [*t*(58) = 4.01, *p* = 0.001, *r* = 0.47] and peri-to-post video [*t*(58) = 7.197, *p* < 0.001, *r* = 0.69]. This was qualified by a day × time interaction [F(2,112) = 3.688, *p* = 0.029, η_p_^2^ = 0.062], with skin conductance rising across all time points but not pre-video to peri-video on *day 8* [*t*(58) = 0.91, *p* = 0.429, *r* = 0.12]. Together, these findings provide no evidence for effects of the intervention on cue reactivity.

### Visual probe

Dwell times were assessed independently for 500-ms trials and 2000-ms trials, as initial and maintained attentional bias can be pharmacologically dissociated in smokers (Freeman et al. 2014). Two (type: smoking-image-containing pairs, neutral pairs) × 2 (target: target image, control image) × 3 (group) mixed ANOVAs were used to assess all eye-tracking data.

Five hundred-millisecond trials showed effects of type [F(1,56) = 5.729, *p* = 0.02, η_p_^2^ = 0.093], target [F(1,56) = 5.295, *p* = 0.025, η_p_^2^ = 0.086] and a type × target [F(1,56) = 7.428, *p* = 0.009, η_p_^2^ = 0.117] and type × target × group interaction [F(2,56) = 3.31, *p* = 0.043, η_p_^2^ = 0.106]. The target × type interaction confirmed the salienceof the smoking cues utilised, evidenced by greater dwell times on smoking target images in the smoking-control pairs [*t*(58) = 3.183, *p* = 0.002, *r* = 0.39] but not in neutral-neutral pairs [*t*(58) = 0.29, n.s]. The type × target × group interaction indicated higher levels of attentional bias in MEM no REACT than PLAC + REACT and MEM+REACT, evidenced by greater dwell time on the smoking target vs. control image in MEM no REACT [t(58) = 3.846, *p* < 0.001, *r* = 0.45], but not in MEM+REACT and PLAC+REACT [*t*s< 1, n.s.]

The 2000-ms dwell times showed type [F(1,56) = 22.706, *p* < 0.01, η_p_^2^ = 0.288] and borderline type × target interaction effects [F(1,56) = 3.891, *p* = 0.053, η_p_^2^ = 0.065], indicating greater overall dwell on to image pairs containing a smoking image and, within these pairs, borderline longer looking at the smoking target image [*t*(58) = 2.01, *p* = 0.05, *r* = 0.25]. First, fixation times showed effects of target F(1,56) = 10.004, *p* = 0.003, η_p_^2^ = 0.152] and a target × type interaction F(1,56) = 6.617, *p* = 0.013, η_p_^2^ = 0.106]. The interaction indicated more rapid fixations on smoking target images than control images [*t*(58) = 5.376, *p* < 0.001, *r* = 0.58], with no difference in initial fixation times in neutral-neutral pairs [t < 0.5, n.s.]. This rapid attentional capture by smoking-related images is indicative of intact motivational salience of these cues.

### Drug blindness check

A chi square of group × participant’s guess on drug (‘don’t know’, ‘drug’, ‘placebo’) found a significant effect of group [χ^2^(4) = 11.74, *p* = 0.019]. Standardised residuals showed that this was driven by fewer participants in the MEM no REACT believing they received the active drug than the other two groups. Guess frequencies are shown in Table 2.

### Manipulation check on reactivation task

All participants in the MMM reactivation conditions correctly recorded the contents of at least three of the six smoking clips. Although the free recall did not explicitly prompt participants to record whether they believed ‘completing the task’ meant smoking a cigarette in the MMM reactivation conditions, all participants in these groups reported that made some reference to their own smoking and 28 of the 39 participants in this group mentioned smoking a cigarette from the box. For the non-reactivation condition, participants again correctly summarised at least three of the scenes. Four participants reported that theyhad been thinking about smoking while watching the control scenes. Re-analysing the data with these participants reclassified as MEM + REACT made no substantive difference to the results. No participants in this group reported that they believed the task would involve smoking.

## Discussion

Employing a translational medicine paradigm with healthy volunteers, we assessed the possibility of inhibiting the reconsolidation of maladaptive cue-smoking memories with memantine in voluntarily quitting cigarette smokers. Ten milligrams of memantine in combination with smoking cue memory retrieval did not significantly impact smoking levels, latency to relapse, craving, cue salience or reactivity to smoking-related stimuli, indicating that memantine did not block the reconsolidation of retrieved cue-smoking MMMs. Indeed, there was some mild evidence of worsening outcomes following memantine, with lower (but non-significant) relapse latency in groups receiving drug. In the group receiving memantine with no memory reactivation, greater attentional bias to smoking cues at test was seen than the groups undergoing memory reactivation with either placebo or memantine. However, the former group experienced higher craving prior to and after capsule treatment and lower belief in receiving the active drug. The observed correlations between pre-reactivation craving, shorter relapse latency, *day 8* smoking and attentional bias suggest that greater tonic craving and reduced expectancy effects in this group may be responsible for this finding. Regardless, memantine with MMM retrieval did not improve relapse latency or smoking outcomes, the clinical outcomes of greatest importance in this study. Thus, no evidence for blockade of MMM reconsolidation by memantine was found in the current study. We believe that these results contribute important insights into priority areas for the successful translation of reconsolidation-based therapies to human addicts.

The high rates of short-latency relapse observed here are typical of smoking cessation and may have masked intervention effects by reducing power to assess long-term group differences. The physiological allostatic drivers of early relapseare likely unaffected by MMM reconsolidation blockade, whereas later, following homeostatic restoration, sensitised mnemonic reward systems play a more significant role in relapse. Reconsolidation-blocking treatments may best employed as relapse-preventing, rather than abstinence-promoting interventions (Milton and Everitt 2012), or may need to be employed in combination with withdrawal management strategies such as nicotine replacement therapy. Given this, it is possible that, had a follow-up session more proximal to intervention been employed (e.g. day 1 + 24 or 48 h), effects of intervention may have been seen. However, the great promise of reconsolidation-based MMM interference lies in its potential therapeutic longevity. Indeed, long-term follow-up periods in smoking studies are the true test of efficacious interventions.

Reconsolidation interference represents the most viable current target for persistently reducing the potency of consolidated MMMs. Previously, attempts to translate preclinical memory-based SUD pharmacotherapies have persevered despite a lack of a cohesive methodological framework or taking account of the methodological shortcomings of previous research (Das and Kamboj 2012; Kamboj et al. 2012; Kamboj et al. 2011), incurring substantial financial and research costs. The methodological and epistemic issues in the current research highlight necessary areas of experimental refinement in response to the observed null results, which, whilst being mindful of clinical relevance, should take precedent in the advancement of this field.

As reconsolidation of drug memories is a ‘silent’ process, only inferred via interference during the reconsolidation window, an epistemological problem exists for null findings which may be attributable to a drug’s inefficacy in interfering with restabilisation, or a lack of memory destabilisation during retrieval. In order to disentangle these, retrieval procedures that consistently destabilise MMMs and alternative compounds that effectively and consistently block restabilisation are required.

In animals, robust blockade of restabilisation of MMMs is achieved using compounds that interfere directly or upstream of neuronal protein synthesis or transcription. This action makes these compounds highly toxic and unsuitable for human use. To date, no drug has shown reliable and lasting reduction in drug use via blockade of MMM reconsolidation in humans. For safety and tolerability, memantine is an attractive NMDAR antagonist for use in the context of interfering with human MMM reconsolidation. However, the current findings do not support this application. Although the current dose was low, memantine (Creeley et al. 2006) shares with other NMDAR antagonists (Das et al. 2013) a complex, non-linear dose-response relationship in mnemonic function implying that optimal dosing for is not simply a case of ‘more-is-better’. Despite this, given the lack of evidence of a memantine effect on almost any measure, it may be that 10 mg was simply too low a dose to observe effects on reconsolidation and it would be prudent to assess higher doses of the drug for reconsolidation blockade. Memantine also has unique kinetic properties at the NMDAR (Black et al. 1996; Blanpied et al. 1997) which may be undesirable in the context of blocking memory restabilisation. In particular, it may not produce the sustained level of NMDAR blockade necessary for disruption in synaptic plasticity during the temporally limited reconsolidation window due to its relatively low affinity, rapid off-rate receptor kinetics (Rammes et al. 2008) and preference for extrasynaptic rather than synaptic NMDARs (Xia et al. 2010).

In contrast, MK-801 (Dizoclipine), the prototypical antagonist for reconsolidation blockade – is paradigmatic with regard to its selectivity, affinity, voltage-dependence and essential irreversibility of blockade during memory destabilisation. The dissociative and psychotomimetic effects are products of the same kinetic profile at NMDARs that cause robust interference with restabilisation, so these effects may be necessary when blocking MMM reconsolidation via NMDARs. While neurotoxicity precludes the use of MK 801 in humans, ketamine may be a realistic alternative. It is approved for human use despite its side effects and already shows some promise for the treatment of SUDs (Krupitsky and Grinenko 1997).

Oral memantine’s slow peak plasma latencymeans it must be administered *prior* to memory retrieval in order to peak post-retrieval. As activation of GluN2b subunit-containing NMDARs is required for memory destabilisation at recall, prior antagonism can *reduce* the ability of memories to destabilise (Mamou et al. 2006), keeping them in a plasticity-resistant state. Given that both groups receiving memantine had slightly (but not significantly) poorer relapse latencies than those receiving placebo, it is possible that this occurred in the current study. Further, NMDAR blockade can engender aberrant prediction error, potentially interfering with successful destabilisation or producing paradoxical effects on memory retention (Corlett et al. 2013). In the current study (and indeed future studies using oral preparations of NMDAergic drugs), this complicates interpretation of findings and may be responsible for observed null findings. However, animal studies have previously administered NMDA antagonists systemically prior to reactivation and shown successful reconsolidation blockade (Milton et al. 2008a; Wu et al. 2012), so pre-reactivation dosing effects likely depend upon locus of administration (Mamou et al. 2006) and selectivity for GluN2b vs GluN2a receptor subtypes (Milton et al. 2013). Dosing after retrieval is ideal as it removes this potential confound. However, for drugs like memantine with long latency to peak activity, this would potentially allow some restabilisation of memory traces before sufficient NMDAR blockade was achieved, reducing the efficacy of the intervention (Milton et al. 2008a; Wu et al. 2012). Ideally, then, NMDARs should be rapidly antagonised, with high receptor saturation, following memory destabilisation. This may preclude the use of oral preparations of NMDAergic drugs for this purpose and will likely require intravenous dosing post-reactivation. If these formulations prove ineffective in reducing MMM strength, NMDAR antagonism may need to be abandoned as a pharmacological target in favour of alternative receptor pathways implicated in memory restabilisation (Blundell et al. 2008; Carrera et al. 2012; de Oliveira Alvares et al. 2008; Makkar et al. 2010). Identifying tolerated pharmacological means for consistently blocking MMM reconsolidation in humans will be key in moving this field forward.

We designed the reactivation procedure used here in an attempt to maximise the potential for memory destabilisation by presenting prototypical smoking cues and engendering uncertainty about reinforcement. This is equivalent to the prototypical reminder without reinforcement in animal reconsolidation studies. We told participants they ‘may or may not be required to complete the task’ (i.e. smoke) following the cue videos, while withholding reward. This aimed to generate prediction error following retrieval, thought to be key in destabilising memories (Sevenster et al. 2013). If however, the reactivation task did not create the expectation of smoking, the possibility of negative prediction occurring would be precluded. Participants’ free-response summary of the reactivation procedure suggests that those in the MMM reactivation groups were thinking about smoking the cigarettes presented in the box. We were, however, unable to collect an independent measure of reward prediction error in the current study; therefore, a potential explanation for the null findings remains.

Given the age and strength (Gräff et al. 2014; Robinson and Franklin 2010) of the smoking MMMs targeted here, it is possible that the reminder structure did not sufficiently destabilise these traces. Although preclinical literature does not always explicitly aim to generate prediction error in reminder procedures, it is likely that PE occurs at retrieval to some extent in preclinical studies, as reminder cues are typically not reinforced during reactivation procedures and learning has generally not reached ceiling level. With the asymptotic levels of learning that are present with MMMs in smoking, prediction error magnitude is retrieval are likely to be low (Schultz et al. 1997) in the absence of procedures that explicitly aim to maximise this parameter.

Alternatively, it is possible that reconsolidation simply does not occur at any meaningful level for memories as strongly encoded as cue-smoking memories in daily smokers. Many researchers have identified the potential of reconsolidation interference for treating SUDs; however, there is a notable paucity of human research directly assessing this. The current research shows that we need to re-assess whether destabilisation of extremely robustly trained MMMs is possible and, if so, what retrieval procedures can reliably produce these effects.

In summary, we found no evidence for 10 mg memantine blocking the reconsolidation of cue-smoking memories in any measure of cue reactivity, craving, salience or relapse in quitting smokers. While memantine in combination with memory reactivation does not appear to be a clinically useful strategy for smoking cessation, the current findings highlight important methodological and epistemological issues in human reconsolidation that must be addressed research to allow the accurate assessment of the clinical potential of post-destabilisation interventions for SUDs.
